# Measuring behavioral and social drivers of COVID-19 vaccination in health workers in Eastern and Southern Africa

**DOI:** 10.1186/s12919-023-00262-1

**Published:** 2023-07-12

**Authors:** Helena Ballester Bon, Symen A. Brouwers, Jenna Mote, Sofia de Almeida, Laurie Markle, Silvia Sommariva, Natalie Fol

**Affiliations:** 1 Eastern and Southern Africa Regional Office, Social and Behavior Change, UNICEF, Nairobi, Kenya; 2 Internet of Good Things, UNICEF HQ, New York, NY USA

**Keywords:** COVID-19, Vaccination, Vaccine demand, Vaccine uptake, Willingness, Social and behavior change, Africa, Internet of Good Things, Health workers

## Abstract

**Background:**

In 2021, twenty out of twenty-one countries in the Eastern and Southern Africa (ESA) region introduced COVID-19 vaccines. With variable willingness to uptake vaccines across countries, the aim of the present study was to better understand factors that impact behavioral and social drivers of vaccination (BeSD). Using the theory-based “increasing vaccination model”, the drivers Thinking & Feeling, Social Processes, Motivation, and Practical Issues were adapted to the COVID-19 context and utilized in a cross-country assessment.

**Methods:**

Data was collected on 27.240 health workers in Kenya, Malawi, Mozambique, South Africa and South Sudan. This was done by administering a survey of seven target questions via the UNICEF Internet of Good Things (IoGT) online platform between February and August 2021.

**Results:**

Findings showed a gap between perceived importance and trust in vaccines: Most health workers thought Covid-19 vaccination was very important for their health, while less than 30% trusted it very much. The pro-vaccination social and work norm was not well established since almost 66% of all respondents would take the vaccine if recommended to them, but only 49% thought most adults would, and only 48% thought their co-workers would. Access was highlighted as a crucial barrier, with less than a quarter reporting that accessing vaccination services for themselves would be very easy. Women exhibited slightly lower scores than men across the board. When testing the associations between drivers in Kenya and South Africa, it appears that when target interventions are developed for specific age groups, social norms become the main drivers of intention to get vaccinated.

**Conclusions:**

The present study revealed various key relations with demographic variables that would help immunization programmes and implementing partners to develop targeted interventions. First, there is a serious gap between perceived importance of COVID-19 vaccines and how much trust people in them. Second, problems with access are still rather serious and solving this would strongly benefit those who demand a vaccine, Third, the role of social norms is the most important predictor of willingness when considering age differences.

**Supplementary Information:**

The online version contains supplementary material available at 10.1186/s12919-023-00262-1.

## Introduction

Twenty out of twenty-one countries in Eastern and Southern Africa (ESA)^1^introduced COVID-19 vaccines in 2021. Despite the compelling evidence on COVID-19 vaccines preventing serious disease, hence reducing hospitalizations and deaths and saving millions of lives, several behavioral and social factors hampered the demand and uptake of vaccines. In a response, the BeSD framework was adapted from existing vaccine literature as a guidance tool to gather and use data that includes individual motivation, trust and confidence in vaccines, barriers in accessing immunization services, and the establishment of positive descriptive social and work norms as drivers of high vaccine uptake [1–3]. These drivers vary across the region in strength and prevalence [4, 5]. Understanding drivers and barriers of vaccine demand and uptake among target groups is essential for reaching and maintaining high coverage of COVID-19 vaccination across the ESA region [6, 7].

Health workers were established as one of the priority groups for the first phase of COVID-19 vaccine roll out [8]. Apart from the heightened risk of COVID-19 infection and transmission in healthcare settings, health workers are also one of the most trusted sources of information on COVID-19 vaccines to the general population and a critical partner in successful demand promotion interventions as well as in the identification and implementation of (community) acceptable demand promotion strategies [9] (cf. [10]).

In the present research, primary analysis utilized health worker based individual-level data from five countries in the Eastern & Southern Africa region. At the time of data collection, COVID-19 vaccine rollout had begun in all surveyed countries except for Kenya, which rolled out vaccination in March 2021. With no vaccinations against COVID-19 in Kenya yet, about 71,000 people nationally were fully vaccinated in South Africa, 410,000 fully vaccinated in Malawi, 5,000 fully vaccinated in South Sudan, and none fully vaccinated in Mozambique [11–14]. No specific data from the time exists on the vaccination amongst although health workers for most of the countries; 57,305 health workers had been vaccinated with one dose in Mozambique. With variation in vaccine uptake across Eastern and Southern Africa, aim of the study was to better understand the social and behavioural drivers (BeSD) that impact the demand of COVID-19 vaccines in selected countries.

## Method

### Participants and procedure

The study utilizes primary analysis of data from 27,240 health workers from five countries in the Eastern & Southern Africa region: Kenya, Malawi, Mozambique, South Africa, and South Sudan. Collection of data took place in Kenya and South Africa in February 2021, in Mozambique in March 2021, and in Malawi and South Sudan in August 2021. The 27,240 health workers come from across the health spectrum and are not specifically limited to the field of immunization.

Surveys adapted to the country context were administered through the Internet of Good Things (IoGT) [15] website. Websites built through IoGT include many customized features that support bridging the digital divide and are by default data-light platforms that function on low-end mobile devices, like feature phones. IoGT-powered websites are used by many UNICEF country offices in ESA, and most are available at no cost to users through mobile network operator zero-rating partnerships. An ad-hoc page with the survey questions was set up on local IoGT sites in all the participating countries.

The 27,240 participants in the present study come from a much larger pool of HWs that were reached out to through a page specifically capturing the COVID-19 vaccination survey for health workers. Links to the survey page were disseminated in collaboration with the respective Ministries of Health (MoH) and partners, for example by texting healthcare workers a promotional message and instructions on how to respond to the survey. The total number of health workers that were initially reached is not known. All participants in the study are HWs who freely volunteered after seeing the messages send to them, following the link contained in the messages. No quality control was able to exist here. The architecture behind the IoGT is based on Microsoft; the data was initially stored here and later downloaded to Excel files for research purposes. Table 1 delineates the full differentiation of the sample by country, age, and gender.

**Table 1:**
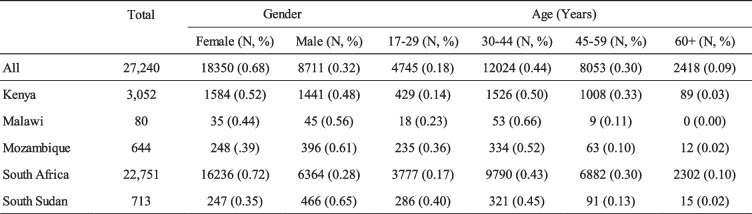
Drivers assessed through the Behavioral and Social Drivers (BeSD) model and their questions

### Measures

**Fig. 1:**
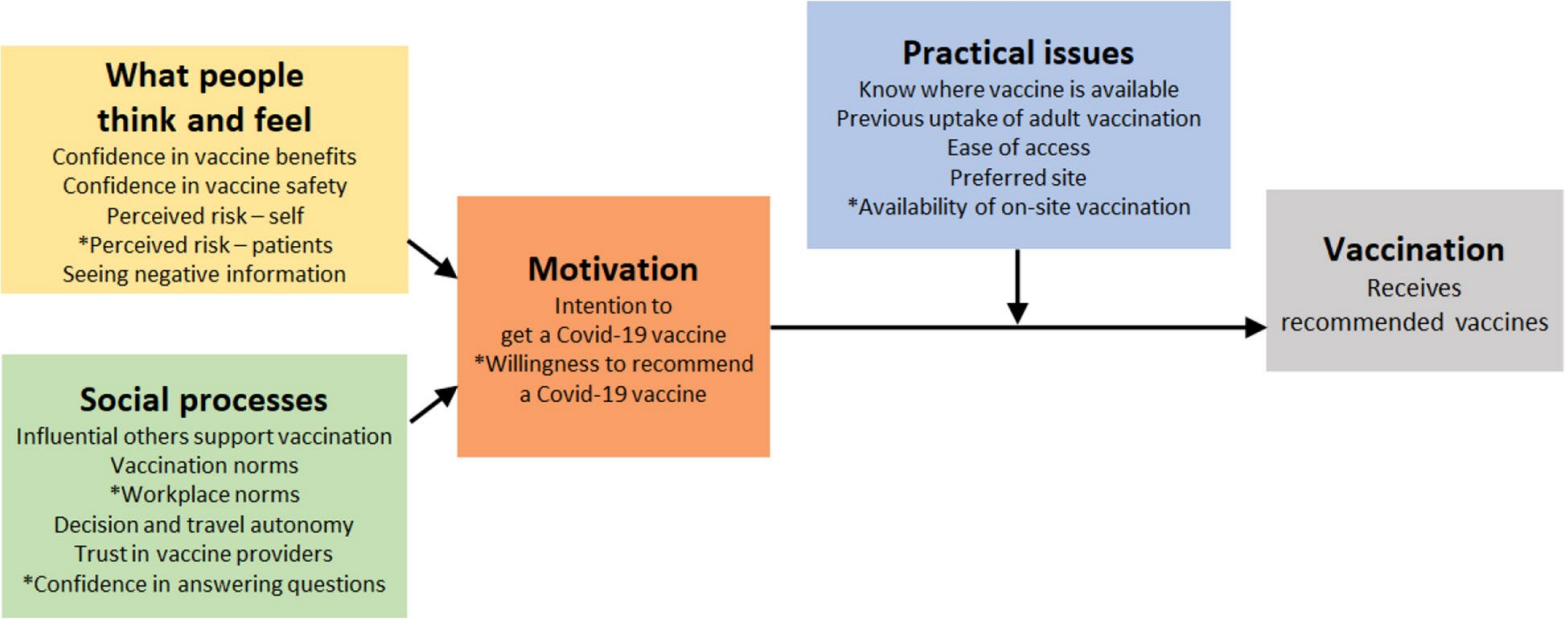
The Behavioral and Social Drivers (BeSD) Framework. Source: Behavioural and social drivers of vaccination: tools and practical guidance for achieving high uptake. Geneva: World Health Organization and UNICEF; 2022

The present study employed selected questions of the survey for health workers to measure all four BeSD drivers (Fig. 1): with seven questions for Kenya, Malawi, Mozambique, South Africa and only five questions for South Sudan (see Appendix). The questions utilized three- or four-point Likert-scales to record the responses of each participant (No, Not sure, Yes, and Not at all, A little, Moderate, and Very much, respectively). The survey sometimes included of variations consisting of two skipped items for South Sudan, for the reason to limit the time taken to complete the survey, and sometimes a slight rephrasing of the questions, without changing substance or intent of the questions.

Social and Behaviour Change (SBC) officers and MoH representatives provided suggestions for phrasing a question or a specific desired topics to assess, so as to adapt the survey to characteristics of the rollout at national level. Table 2 exhibits the administered questions by their applicable drivers [16]. In addition to the core questions of the BeSD, several questions were included to assess gender, age, health worker role, and geographic location. See Appendix A for the full questionnaire.

**Table 2:**
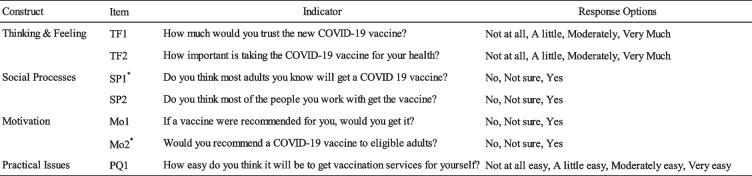
Sample composition by country and gender. [Note: Items SP1 and Mo2 were omitted in South Sudan]

### Data-analysis

Post-stratification weights were calculated to enable the development of frequencies that are more representative for the general health worker population, in each respective country. Sample ratios were generated for gender, age, and geographic location, and matched with 2020 population ratios, derived from the Organization for Economic Co-operation and Development (OECD), World Bank, and local census data [17–19]. At an individual, case-based participant level, the ratios led to population/sample weights for each demographic. A single overall weight was calculated as a product of the three demographic weights and split under each variable by answer options.

Percentages by item were separately extracted in the statistical analysis software platform SPSS, by gender, country, and combination of gender and country. Each percentage was then multiplied by its applicable weight to establish the weighted percentages.

**Fig. 2:**
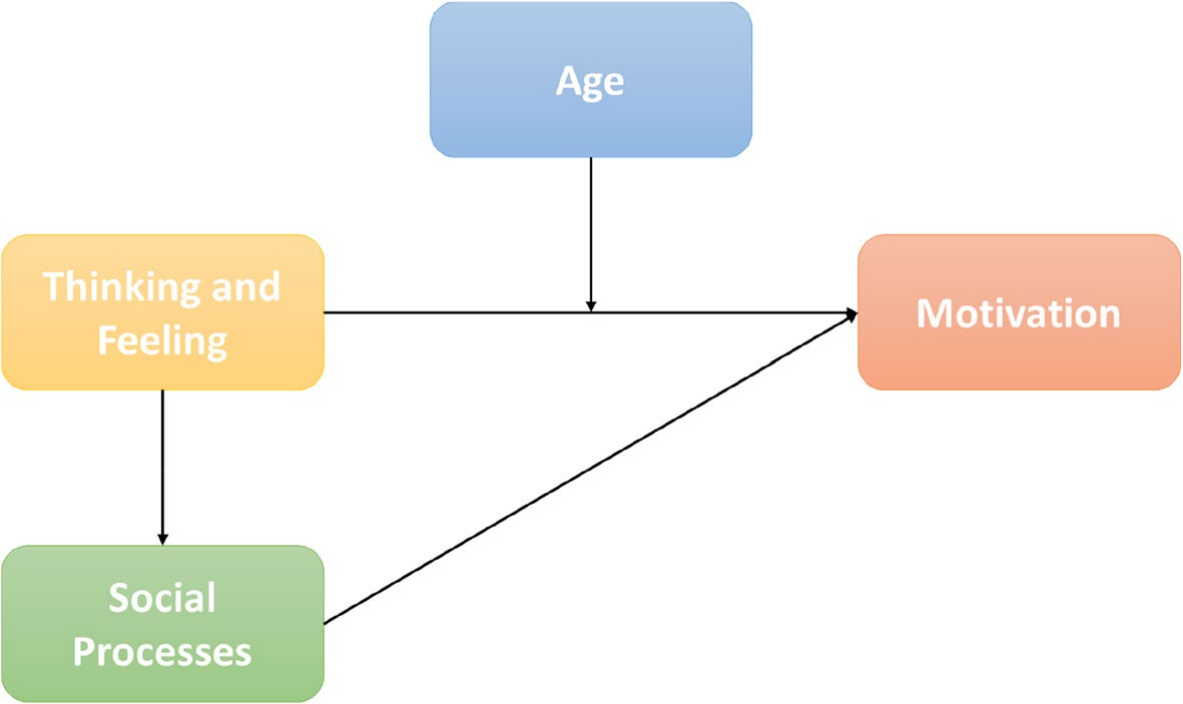
The theoretical BeSD model with the hypothesizes paths between Thinking & Feeling, Social Processes, and Motivation. [Note: Each path reflects an association between antecedent and consequent (for example from Thinking & Feeling to Social Processes), with analysis devoted to establishing the directionality of the path (positive, zero, negative) and size of the association. Thus, the analysis answers, for example, [if..] Thinking & Feeling receives higher scores, will Social Processes [..then] also increase, or perhaps decrease – and by how much]

Path analysis to test correlations between drivers influencing vaccine demand was conducted in the structural equation modeling software platform AMOS on unweighted data. A confirmatory structural equations model was created with Thinking and Feeling, Social Processes, and Motivation as three latent variables, with the respective observed indicators from the data as observed variables. A graphical depiction of the path model is visible in Fig. 2. Practical Issues was omitted from the model as it was assumed for the purpose of this exercise that this variable only enters the picture at a later stage in the context of vaccine uptake.

Multigroup analysis was employed to determine the level of comparability and stability of patterns in the data. Criteria used to evaluate data-fit of the model were CMIN/df, CFI (> 0.95), TLI (> 0.95), RMSEA (< 0.06)), and the SRMR (< 0.08) [20]. For the comparison of multigroup models with nested constraints (i.e., same visual structure but different size values for the paths) the AIC change was used. In a first step, the model is tested separately in South Africa and Kenya, the countries with the largest sample sizes. In this step, it is tested whether the model “works” in South Africa and Kenya. In a second step, age was added as a moderator to Thinking & Feeling. Again, this new model was tested separately in South Africa and Kenya, to see if the addition of age constituted an improvement in each. In a third step, South Africa and Kenya were examined simultaneously as two separate groups, allowing a direct comparison of the various elements in the model across the two countries.

## Results

### Descriptives

Table 2 outlines the composition of the sample. Of the sample total, 18,350 participants in the study were female (67.36%). The study sample showed a wide dispersion in age, with most respondents in the range of 30 to 44 years (44.14%) and 45 to 59 years (29.56%).

The weighted frequencies are presented in two tables by age, gender, and country to search for patterns across the various demographics. Table 3 captures the weighted frequencies by age and gender, Table 4 the weighed frequencies by country and gender.

**Table 3:**
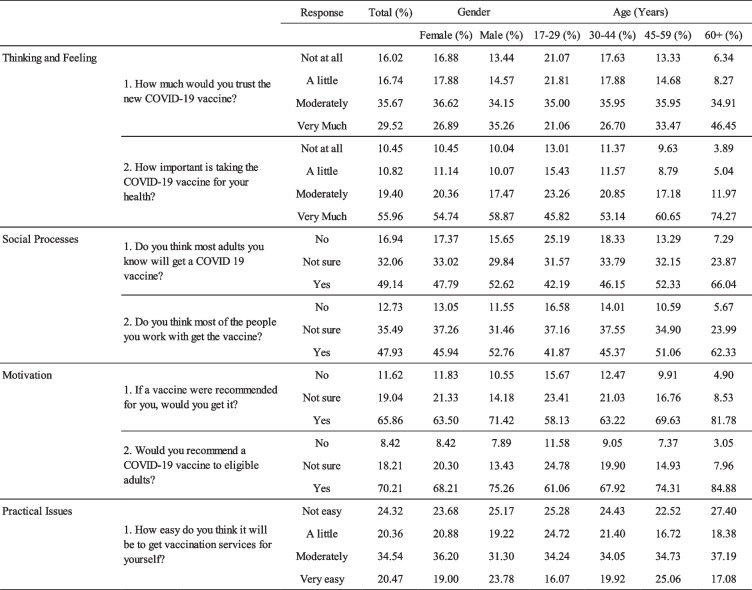
Behavioral and social driver frequencies by age and gender. [Note: The two outermost left columns capture the relevant driver and item. In the third column the response option for each item is captured. Each data entry is a frequency out of 100%, so that within each item by a selected group the four frequencies count to 100%]

**Table 4:**
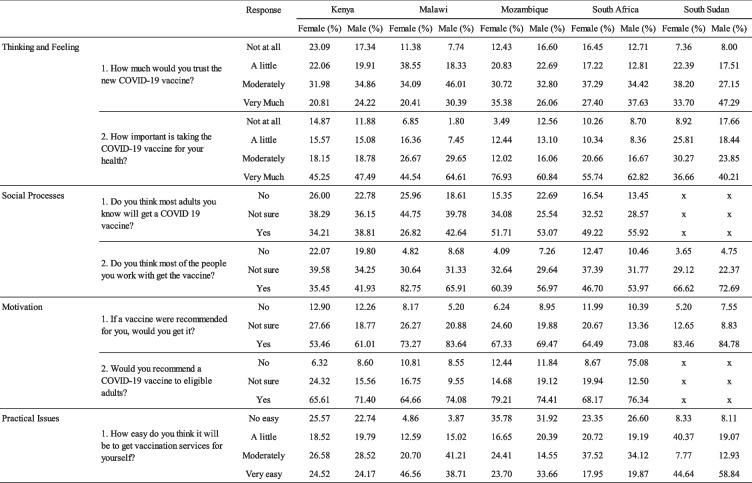
Behavioral and social driver frequencies by country and gender. [Note: The two outermost left columns capture the relevant driver and item. In the third column the response option for each item is captured. Each data entry is a frequency out of 100%, so that within each item by a selected group the four frequencies count to 100%]

For gender, in Table 3 it can be found that for all questions, women provided slightly higher frequencies than men on answer options that indicated less trust, less confidence, less likeliness to give a recommendation, and less ease of access. Equally across both genders, people tended to respond more positively than negatively on all the drivers, the only apparent exception for Practical Issues, where both genders responded more negatively about ease of access.

Across age, various patterns can be observed in Table 3. First, looking down across answer options within each age group and across age groups and for most variables, each subsequent step in more positive response (e.g., from 'moderately' to 'very') had an increased frequency. Negative item responses declined in frequency as age increased. Second, from left to right across age groups in each answer option, for negative item responses frequencies declined, and within more optimistic responses (e.g., “Moderately”, “Very much”) frequencies increased.

This data showcases that respondents from all age groups shared more positive than negative responses across drivers, and that on Thinking & Feeling, Social Processes, and Motivation, the group with people over 60 years of age showed more positive responses than younger respondents. The exception is Practical Issues, which appears to stay more or less stable across age.

In addition to general patterns for age and gender, various more specific contrasts highlight the presence of barriers or clear reasons why people do not take up vaccines more. For example, a great deal of respondents thought a vaccine was very important for their health, but much less people have trust in it (55.96% vs. 29.52%). There is also a key juxtaposition between self and other visible in the data: Respondents are more positive about their own willingness than they are about other taking up the vaccine (65.86% vs. 49.14% for other adults and 47.93% for co-workers). In general, people are negative about the ease of uptake, with only 20.47% thinking it would be easy.

An identifiable contrast that does show is generally more negative attitudes and perceptions in Kenya across all questions. In South Sudan, intention to vaccinate was the highest (83.46% for women, 84.78% for men). It also appears women in Mozambique had the highest frequency of those reporting that the vaccine is very important for their health (76.93% reported the vaccine is very important compared to 3.49% that see no importance at all.)

We can see that in most countries (Table 4), men responded more positively than women across the board, but that in Mozambique the reverse seems to be the case, with men responding more negatively than women – except for Practical Issues, where responses from men behave more in line with the general patterns.

### Model testing

Tests highlighted the contribution of age-related moderators to social norms and willingness to uptake vaccines and the differences and similarities between Kenya and South Africa in terms of these effects. Indices specifying the fit of the model to the data are presented in Table 5.

**Table 5:**
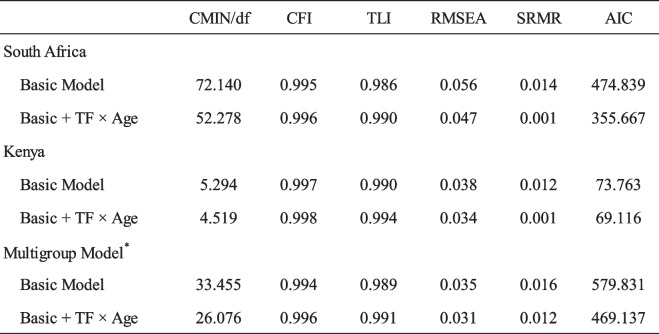
Fit indices from the multigroup structural equation modeling. [Note: *Indices for the best fitting model printed here only]

**Fig. 3:**
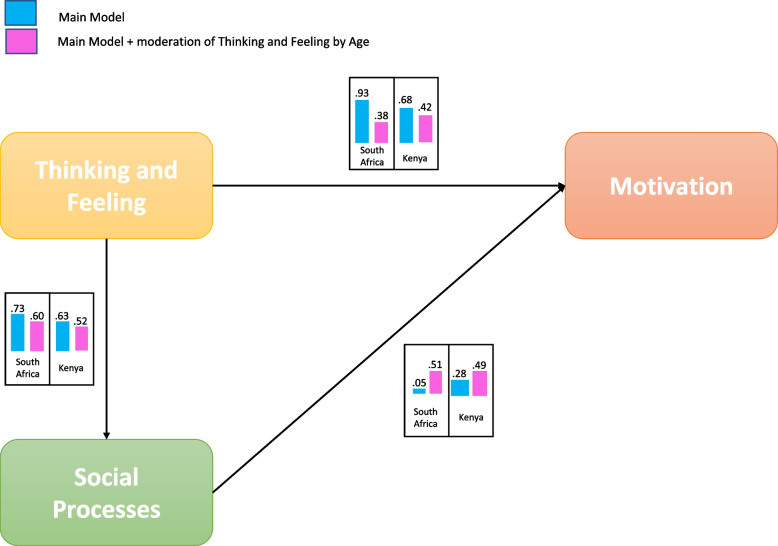
The tested BeSD model with age as a moderator showing values for each path in South Africa and Kenya

From the indices it can be gleaned that all six models fit the data well. However, from the change in AIC (474.839—355.667 in South Africa, and 73.763 – 69.116 in Kenya) it is clear that the addition of age as a moderator of Thinking & Feeling presents a significant improvement in fit. The effects of adding age as an extra parameter in the model are displayed in Fig. 3.

In South Africa, while initially Thinking & Feeling had the primary effect on Motivation (β = 0.93), with little or no effect from Social Processes (β = 0.05), the specification of potentially distinct mechanisms of Thinking & Feeling at different age groups, brings out the effects of Social Processes (β = 0.51). In the Kenya dataset a similar addition of age to the model could be found (the link between Social Processes and Motivation increases from β = 0.28 to β = 0.49). From this finding it is thus possible to conclude that when we develop target interventions for specific age groups, social norms become the main drivers of intention to get vaccinated.

The final multigroup comparison of South Africa and Kenya repeats the finding that age is a strong addition for moderating the effects of Thinking & Feeling, but also that the paths between the three BeSD are similar in South Africa and Kenya: When we look at models with different assumptions about their similarity across cultures^2^, the test shows that the model with the same values for the paths gives the best fit. The path from Thinking & Feeling to Motivation is β = 0.39, and from Social Processes to Motivation β = 0.50.

## Conclusions

The present study revealed various key relations with demographic variables that would help immunization programmes and implementing partners to develop more targeted interventions. First, for all behavioral and social drivers in the BeSD framework, women provided higher frequencies than men on answer options that indicated less trust, less confidence, less likely to give a recommendation, and less ease of access. Overall, though, people tended to respond more positively than negatively on all the drivers, and this is for both women and men, the only exception for Practical Issues, where both genders responded more negatively about ease of access. Age also had a major impact on the results, which could be seen in the numbers on prevalence, where with increasing age, each next step in more positive response (e.g., from 'moderately' to 'very') had an increased frequency. Also, negative item responses declined in frequency as age increased. More substantially, structural equation modelling in Kenya and South Africa showed that age shapes the impact of social norms on willingness to vaccinate. While a gap in social and workplace norms was observed for female and younger respondents in this sample, social norms did emerge as a strong precursor for motivation in the present study.

Considering the intention to vaccinate is high in the region, demand promotion interventions should focus on activating the intentions of those who do want to be vaccinated to reinforce positive social norms, while continuing to target trust-building interventions toward those who are hesitant. Practical Issues was found to be a critical dimension to consider, as despite respondents being all health workers, many reported perceived lack of ease to access vaccination services for themselves. Findings highlight the need to get a better understanding of how Practical Issues affect the intention-action gap. Evidence-based advocacy may be necessary to appropriately adapt service delivery to reduce access barriers, simplify registration mechanisms, and ensure people know when, where, and how to be vaccinated.

The analysis also showed a dissonance between confidence in the benefits and vaccination and trust in the vaccine amongst health workers in five countries in Eastern & Southern Africa. While most health workers had positive attitudes toward vaccination (COVID-19 vaccines are effective, prevent disease, save lives etc.), less of them thought that the vaccine is safe and not dangerous or harmful—reinforcing the need for trust-building interventions.

The primary key to the proper functioning of Thinking & Feeling and Social Processes in the context of vaccination demand was age. Given that the effects of Thinking & Feeling are moderated by age, and that the inclusion of age in the model strengthened the effect of Social Processes on Motivation, it is important to consider targeted interventions for different age groups, particularly around Thinking and Feeling. In Kenya and South Africa these mechanisms between the variables in the model are also remarkably similar, indicating strong robustness in the way age works between Thinking & Feeling and Social Processes. Differences between the two countries are limited to the small size of effects.

Various parallels with existing literature on age and gender, in relation to behavioral and social drivers, can be observed. Considerations related to COVID-19 vaccines for women and children are “particularly difficult… given the low availability of COVID-19 data relevant to these populations” [21]. For example, the notion that people of different ages realize different social accomplishments and respond to different social challenges, is captured in the social stage theory of Erikson (1959), with adolescents and young adults more involved with forming their identities and older adults more with generativity. They are susceptible to different types of inputs and need to be engaged in different ways. Another parallel with recent literature on COVID-19 vaccination from the American South, however, is that younger adults are more resistant to the vaccine than older [22]. Reaching children and adolescents (CA), has strong benefits, though, in “mitigating educational disruptions associated with school closures, helping to maintain the continuity of feeding schemes, and limiting disruptions to extracurricular activities. Providing COVID-19 vaccines to CA may help to minimise the cascading social and economic impacts that COVID-19 infections among CA could have on parents, families and communities” [23].

Pregnant women in Africa are among the most vulnerable of groups. Consistently reporting less trust, less confidence, less likely to give a recommendation, and less ease of access, by women as to men is linked by several researchers to a so-called infodemic [24]. Investment in partnerships with women’s, youth, and other community-based organizations can strengthen interventions to promote, facilitate and sustain COVID-19 vaccine uptake. Linkages between Thinking and Feeling drivers with the level of exposure to social media and infodemics can be explored, and targeted interventions and trusted channels for specific age groups should be considered.

It is important to note a few limitations to this study. First, these surveys were undertaken when few people had been vaccinated and before emergence of the Omicron variant, which increased public concerns on effectiveness [25]. Second, the unavailability of information on the total number of health workers in each country makes it challenging to put the data in the context of the overall healthcare workforce and investigate generalizability. Third, in the descriptive narrative of findings, it is important to interpret differences between frequencies of the different responses (e.g., “a little”, “moderately”) with caution, as people from different groups (age, gender, country) possibly attach different meaning to these responses. Fourth, in the survey design, inclusion of more questions per driver would have enhanced the study and testing of the model but was not possible due to practical constraints in the survey implementation. Fifth, the lack of control by researchers over whom visits the IoGT site with the present survey advertised there and whom of those visitors decide to enter the survey, might pose threats to sampling representatives. This threat was (partially) addressed by using the national governments and MoHs to reach as many people as possible. Sixth, it is possible that social desirability lead respondents to answer is distinctive ways, such as extreme scores (very low, very high), or agreeing (always saying yes). There is no exact way to verify these biases, but the scores are substantially varied across answer options.

Age and gender are particularly sensitive demographics that shape and guide how interventions are received. Targeting programs specifically to women and people of different ages will best the most effective to reach high vaccine demand.

### Supplementary Information

**Additional file 1:** **Appendix A.** The BeSD of COVID-19 vaccination survey guide from Data for Action: Achieving high uptake of COVID-19 vaccines.

## Data Availability

The data that support the findings of this study were used under license for the current study, and so are not publicly available. However, data are available from each country’s IoGT focal point upon reasonable request and with prior permission of the Department of Health, where applicable.

## Abbreviations

BeSD: Behavioral and Social Drivers
COs: Country Offices
COVID-19: CO_VI_D-19: Corona Virus Disease 2019
ESA: Eastern and Southern Africa
IoGT: Internet of Good Things
MoH: Ministry of Health
OECD: Organization for Economic Cooperation and Development
SBC: Social and Behavioral Change
SB: Social and Behavioral
WHO: World Health Organization
UNICEF: United Nations Children's Fund

## Footnotes

1 Angola, Botswana, Burundi, Comoros, Eswatini, Ethiopia, Kenya, Lesotho, Madagascar, Malawi, Mozambique, Namibia, Rwanda, Somalia, South Africa, South Sudan, Tanzania, Uganda, Zambia and Zimbabwe.

2 A. same visual structure but different size values for the paths, B. same value for the paths, and C. most restrictive with same exact mean values for the variables in each country.
