# Supplementary material for: Measuring behavioral and social drivers of COVID-19 vaccination in health workers in Eastern and Southern Africa
Source: BMC Proc. 2023 Jul 12;17(Suppl 7):14. doi: 10.1186/s12919-023-00262-1 (PMC10337050; doi:10.1186/s12919-023-00262-1)
Supplement: Supplementary file 1 — Additional file 1: Appendix A. The BeSD of COVID-19 vaccination survey guide from Data for Action: Achieving high uptake of COVID-19 vaccines. [file 12919_2023_262_MOESM1_ESM.docx]

Appendix A

The BeSD of COVID-19 vaccination survey guide from Data for Action: Achieving high uptake of COVID-19 vaccines (WHO and UNICEF, 2021).

*Survey and Question Rationale*

The table below compiles survey questions for both adults and health workers. Table cell colours are indicative of the domain (thinking and feeling, social processes, motivation and practical issues).

A total of 21 BeSD questions apply to adults, and up to 28 BeSD questions can apply to respondents who are health workers. To supplement the BeSD COVID-19 questions, included below are also recommended consent script (S0) with adaptable fields for countries to modify and use as appropriate, and six socio-demographic questions (S1–S6) for country adaptation. These supplementary questions (S0–S6) are considered the minimum necessary for quality data collection and analysis. These can be used and adapted as needed to support the research objectives.

Researchers may also carefully choose to add specific new socio-demographic questions to support more granular interpretation of the data. The survey flow adopts the logic of “facts” before “attitudes”, and “attitudes” before “intentions”, and moves from general immunization questions to COVID-19 vaccine specific questions. The column “item rationale” contains important information for translation and local adaption of questions, including how to adapt questions for post-vaccine introduction.

Wording in [square brackets] is to indicate terminology that will likely need to be locally adapted. Text all in CAPITALS is an instruction for the interviewer and must not be read aloud for participants.

|  | **Driver** | **Question rationale** | **Adult question** | **Health worker question** |
| --- | --- | --- | --- | --- |
| S0 | **Consent** | This question serves as an example of text to be included to capture respondent’s informed consent to their participation in the study.  The wording in [square brackets] can be adapted at the local level to reflect accurate information in the relevant fields.  Text in ALL CAPITALS is an instruction for the interviewer and must not be read aloud for participants. | Hello, I am [INTERVIEWER’S NAME] with [INSTITUTION OR ORGANIZATION NAME]. We are interviewing people to help improve vaccination services in [NAME OF COUNTRY].  I know you are busy, so this will take only a few minutes. Your participation is completely voluntary and anonymous. If you do not want to answer a question or wish to stop the interview, just let me know.  Would you be willing to take the survey?   - Yes - No   IF “YES” TO S0: Thank you very much. Do you have any questions for me before we begin?  PROCEED TO SURVEY SCREENER AFTER ADDRESSING ANY QUESTIONS.  IF “NO” TO S0: Thank you very much. END INTERVIEW. | [same as Adult] |
| S1 | **Age** | Question collects age in number of completed years, this will allow for stratified analysis by age of respondents. This question can also serve to screen in or screen out participants for inclusion based on the study sampling methodology. | How old are you?  _________ years | [same as Adult] |
| S2 | **Gender** | Question collects gender identity of respondents to allow for stratified analysis. The third response option can be included in contexts where specific third gender categories are culturally recognized; this response option can be adapted as appropriate based on in-country considerations or consultation. | What is your gender?   - Woman - Man - Non-binary or transgender - Prefer not to say | [same as Adult] |
| S3 | **Occupation** | This question enables sorting of respondents for the right survey as needed. Inclusion of this question will allow analysis for intentions to be stratified by whether someone is a priority occupational group or not.  This question can also serve to screen in or screen out participants for inclusion based on the study sampling methodology.  “Essential services worker” refers to other non-health frontline workers (e.g., police, transport service workers, grocery store staff, etc.).  The categories may be locally adapted to ensure they are appropriate to the specific context and allow for disaggregated data as needed. Some countries may choose to delineate between frontline and non-frontline health workers. | Which of the following best describes your work during the COVID-19 pandemic?   - Health worker - Essential services worker - Educator - Other worker - Not currently in paid work - Retired - None of the above | [same as Adult] |
| S4 | **Health worker role** | This question allows for categorization of health workers into common roles or functions within the health system. If included, this question enables more detailed analysis of health worker role and stratification of results.  The response options offered should be adapted in-country at national or even subnational level to reflect the most appropriate role categorizations based on the types of health workers most likely to be at risk of COVID-19 infection/most exposed to COVID-19. | n/a | What is your current role?   - Doctor - Nurse - Paramedic/first responder - Allied health - Community health worker - Traditional healer - Other health worker |
| S5 | **Comorbidities or underlying conditions** | This question assesses whether the respondent has any underlying illness, comorbidities or health conditions that make the respondent a higher priority for vaccination. Inclusion of this question would allow for stratification of results by comorbidities.  This question can also serve to screen in or screen out participants for inclusion based on the study sampling methodology. | Do you have a chronic illness?   - Yes - No - Not sure | [same as Adult] |
| S6 | **Previously diagnosed with COVID-19** | Previous infection with COVID-19 can be perceived as a reason to not vaccinate, and countries may want to stratify data on intentions to be vaccinated according to this. This question can also serve to screen in or screen out participants for inclusion based on the study sampling methodology.  When a COVID-19 vaccine becomes available in-country, researchers may choose to include a question to assess whether the respondent has received a COVID-19 vaccine. If several are available in the country, a question to ask which vaccine the respondent received may also be added. | To your knowledge, are you, or have you been, infected with COVID-19?   - Yes - No   IF “YES”:  Was it mild or severe?   - Mild - Severe   Was it confirmed by a test?   - Confirmed by a test - Not confirmed by a test | [same as Adult] |
